# Could Omega 3 Fatty Acids Preserve Muscle Health in Rheumatoid Arthritis?

**DOI:** 10.3390/nu12010223

**Published:** 2020-01-15

**Authors:** Kassandra Lanchais, Frederic Capel, Anne Tournadre

**Affiliations:** 1 Université Clermont Auvergne, INRAE, Unité de Nutrition Humaine (UNH), 28 Place Henri Dunant—BP 38, UFR Médecine, UMR1019, 63009 Clermont-Ferrand, France; kalanchais@hotmail.fr (K.L.); atournadre@chu-clermontferrand.fr (A.T.); 2 CHU de Clermont-Ferrand, Service de rhumatologie, 63003 Clermont-Ferrand, France

**Keywords:** rheumatoid arthritis, cardiovascular diseases, muscle, lipids, omega 3

## Abstract

Rheumatoid arthritis (RA) is a chronic inflammatory disease characterized by a high prevalence of death due to cardiometabolic diseases. As observed during the aging process, several comorbidities, such as cardiovascular disorders (CVD), insulin resistance, metabolic syndrome and sarcopenia, are frequently associated to RA. These abnormalities could be closely linked to alterations in lipid metabolism. Indeed, RA patients exhibit a lipid paradox, defined by reduced levels of total, low-density lipoprotein (LDL) and high-density lipoprotein (HDL) cholesterol whereas the CVD risk is increased. Moreover, the accumulation of toxic lipid mediators (i.e., lipotoxicity) in skeletal muscles can induce mitochondrial dysfunctions and insulin resistance, which are both crucial determinants of CVD and sarcopenia. The prevention or reversion of these biological perturbations in RA patients could contribute to the maintenance of muscle health and thus be protective against the increased risk for cardiometabolic diseases, dysmobility and mortality. Yet, several studies have shown that omega 3 fatty acids (FA) could prevent the development of RA, improve muscle metabolism and limit muscle atrophy in obese and insulin-resistant subjects. Thereby, dietary supplementation with omega 3 FA should be a promising strategy to counteract muscle lipotoxicity and for the prevention of comorbidities in RA patients.

## 1. Introduction

Rheumatoid arthritis (RA) is the most prevalent inflammatory rheumatic disease, affecting 0.5% to 1% of the world’s population, mainly women (sex-ratio 3 women: 1 man) [1]. This autoimmune disease induces joint destructions, functional impairment, disability, and an excess of mortality [2]. The pathogenesis of the disease is complex, including genetic susceptibility (Human leucocyte antigen DRB1 allele (HLA-DRB1) and shared epitope), epigenetic modifications and environmental factors (psychologic stress, smoking, obesity, microbiota…). RA is characterized by a loss of tolerance with subsequent inflammatory infiltration of the synovial membrane and the production of autoantibodies against citrullinated peptides (ACPAs) and IgG (rheumatoid factor [RF]) [3]. The inflammatory infiltrates in synovial membrane include monocytes, T lymphocytes (Th1 and Th17) and B cells which are responsible for the production of proinflammatory cytokines (TNF-α, IL-1β, IL-6) and chemokines, the overexpression of adhesion molecules leading to a self-sustained loop by inducing and aggravating the immune process, attracting immune cells, and activating osteoclast and the degradation of the cartilage matrix. RA patients present morning joint stiffness and swelling, systemic inflammatory response (elevated erythrocyte sedimentation rate and C-reactive protein) and autoantibodies (RF and ACPAs). Therapeutics include synthetic and biologic disease-modifying antirheumatic drugs (DMARDs), targeting inflammation and the progression of structural damages. Treatment is usually initiated with methotrexate, the most frequently used conventional synthetic DMARDs, in association with a low dose of glucocorticoid that should be tapered and then stopped within 6 months. In case of refractory disease, biological therapeutics such as inhibitors of TNF-α, IL-6, T-cell co-stimulation or B cell depletion, can be used. More recently, targeted synthetic DMARDs against janus kinase (JAK) have been developed to modulate cytokine intracellular signaling.

RA patients have a greater cardiovascular (CV) risk, more metabolic disorders such as insulin resistance and sarcopenia associated to increased intramuscular fat in comparison to healthy subjects [4]. An accumulation of scientific evidences suggests that diet could play a role in the prevention of this chronic disease. While it has been demonstrated that saturated FA (SFA) promote inflammation and insulin resistance, omega 3 fatty acids (FA) are crucial components of a healthy diet. Eicosapentaenoic acid (EPA) and docosahexaenoic acid (DHA) were shown to prevent the development of inflammation [5,6], cardiovascular diseases (CVD) [7,8], lipotoxicity [9] and metabolic abnormalities in skeletal muscle [10]. Only few studies have investigated the effect of omega 3 FA on muscle health in the context of chronic inflammation associated to a loss of muscle mass and function such as RA.

Thus, the aim of this review was to illustrate the potential effect of omega 3 FA on metabolic disorders and skeletal muscle’s abnormalities in the context of RA.

## 2. Cardiometabolic Comorbidities in Rheumatic Diseases

### 2.1. Cardiometabolic Comorbidities in RA

**Figure 1:**
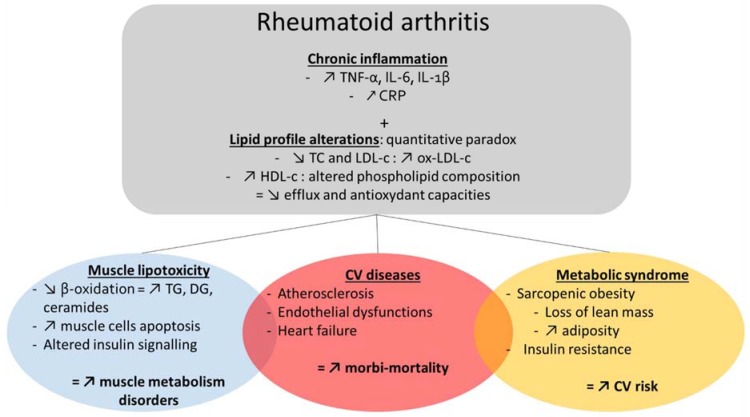
Comorbidities in rheumatoid arthritis (RA). RA is a chronic inflammatory disease characterized by alterations in lipid profile, a high prevalence of metabolic syndrome, sarcopenic obesity, insulin resistance, altered insulin signaling and disorders in muscle lipid metabolism. All of these perturbations contribute to CV disease and thus, to an increased morbi-mortality in RA patients. CRP: C—reactive protein; CV: Cardiovascular; DG: Diglycerides; HDL-c: High-density lipoprotein-cholesterol; IL-6: interleukine-6; IL-1β: interleukine-1β; LDL-c: Low-density lipoprotein-cholesterol; ox-LDL-c: oxidized low-density lipoprotein-cholesterol; TC: Total cholesterol; TG: Triglycerides; TNF-α: Tumorous Nuclear Factor α.

CVD are the major cause of death in RA patients [11]. Indeed, one half of RA patients die of CVD [12]. The risk of CVD is then increased by 48% in RA patients and was shown to be markedly raised in women before the age of 50 years old [11]. RA induces an accelerated atherosclerosis and several studies demonstrated subclinical atherosclerosis in inflammatory arthritis with altered vascular function and morphologic changes. Endothelial dysfunction was observed concomitantly to increased arterial stiffness and carotid intima-media thickness [11]. Traditional CV risk factors, such as smoking, hypertension, diabetes, hypercholesterolemia, obesity do not fully explain the increased CV risk in RA patients. The excessive CV risk persists after adjusting for traditional CV risk factors [12]. Inflammation promotes atherogenesis, exacerbates the established CV risk factors and may then account for this effect. Proinflammatory cytokines such as TNF-α, IL-1, IL-6, IL-17 contribute to the formation and the instability of the atherosclerotic plaque that is at least partly due to an alteration of the lipid structure and function (Figure 1) [11,13,14,15,16,17]. The association of a high CRP level with CV risk factors and its direct contribution to atherothrombosis remains controversial. However, elevated CRP levels were shown to increase by two-fold the risk of coronary artery disease in RA patients without diabetes mellitus or metabolic syndrome [18]. In addition, The Canakinumab Anti- inflammatory Thrombosis Outcome Study (CANTOS) showed the beneficial effect of reducing inflammation on CV events through IL-1 inhibition. The treatment of patients with previous myocardial infarction and a high CRP level, with a monoclonal antibody targeting IL-1β (canakinumab) reduced the recurrence of major cardiac events supporting the inflammatory theory of atherosclerosis.

Obesity and metabolic syndrome are also well recognized risk factors for CV diseases and all-cause mortality [19]. Insulin resistance and alterations in body composition, including an increase in visceral adipose tissue and decrease in lean mass are all key characteristics of RA patients that could contribute to cardiometabolic disorders. The prevalence of insulin resistance is increased by 31% and the risk of diabetes by approximately 50% in RA patients compared to healthy subjects matched for age and sex [13,20,21] (Figure 1). In RA patients, high visceral adipose tissue was associated to elevated fasting glucose, hypertension, metabolic syndrome [22,23].

RA patients are 3-fold more sarcopenic than healthy controls [24] and cachexia-associated metabolic disorder could explain the fact that a low body mass index (BMI) has been associated with cardiovascular death in RA patients [25]. Body fat is increased in RA patients with normal BMI [26,27], which led to define the sarcopenic obesity phenotype by a decrease in lean mass while fat mass may be preserved or even increased regardless of changes in total body weight. Alterations in muscle density, ectopic fat accumulation were also observed during sarcopenic obesity [28,29,30]. These abnormalities contribute to the alteration of muscle performances, inducing frailty and disability in RA patients, which, in turn, exacerbate muscle loss and fat deposition [4,31,32]. Moreover, epicardial adipose tissue is increased in RA and produces pro-inflammatory cytokines responsible of an early alteration of myocardial functions, thus leading to an increased risk of CVD [33,34]. Yet, higher visceral fat and a low BMI in patients with RA are implicated in the increased CVD risk (Figure 1) [22,25]. Thus, it is essential to fight against sarcopenic obesity and insulin resistance to improve patients’ quality of life and counteract excess mortality due to RA.

### 2.2. Cardiometabolic Comorbidities in Other Rheumatic Diseases

Cardiometabolic comorbidities and alterations of body composition were also observed in osteoarthritis (OA) and psoriatic arthritis (PsA).

Sarcopenia and obesity were frequently reported, affecting 22% to 30% of the patients with OA [35,36]. An increased risk of myocardial infarction, stroke and metabolic syndrome was also reported as well as atherogenic lipid profile, elevated fasting glucose [37]. Metabolic syndrome, CV disease and OA are then closely related. Obesity and fat mass contribute to mechanic loads on the joints. A sedentary lifestyle, low-grade inflammation with the production by the adipose tissue of proinflammatory mediators (cytokines, adipokines, FA, and reactive oxygen species (ROS)) and the development of metabolic syndrome with all its components (diabetes, dyslipidemia, and/or hypertension) are, in turn, risk factors for OA [38].

In contrast to OA, PsA is an inflammatory rheumatic disease. Few data on body composition and CV risk are available in comparison to RA. However, obesity and sarcopenia are also frequently reported [39,40]; CV risk is increased, although less established than in RA. Due to the high prevalence of obesity and type 2 diabetes, PsA is considered to be more related to metabolic comorbidities than RA [41].

Thus, common pathophysiological pathways (low or high-grade inflammation, insulin resistance, lipotoxicity, mitochondrial dysfunction) are possibly involved in different degrees in RA, OA and PsA. Inflammation is the key factor in the development of cardiometabolic disorders in RA. Adiposity is associated with metabolic OA while PsA combines chronic inflammation and metabolic disorders (obesity, alteration in lipid homeostasis, insulin resistance) with probably a greater impact of metabolic syndrome on CV risk.

## 3. Alterations of Lipid Profile: A Mutual Denominator of Comorbidities in RA

### 3.1. Altered Lipidome in RA

Several studies have demonstrated the existence of a lipid paradox in RA patients. Indeed, total cholesterol and low-density lipoprotein cholesterol (LDL-c) are decreased, while CV risk is increased [42,43,44]. However, even if LDL-c is decreased, the accumulation of oxidized-LDL-c contributes to CV risk in RA patients [45]. In addition, the alteration in the composition of high-density lipoprotein (HDL) phospholipids could be responsible for a decrease in atheroprotective function and has also been proposed to explain the higher CV risk in RA. A lipidomic signature was identified in the HDL from RA patients, with a modification of the lipidome of small HDL and a reduction omega 3 FA in HDL phospholipids compared to control subjects [16,46]. This could be associated to an increase in phospholipase A2 activity, leading to the alteration of phospholipid profile and thus, to a reduction of efflux and antioxidant capacities of the HDL and finally, to an increased risk for CVD [43,47].

### 3.2. Metabolic Disorders and Sarcopenia in RA

Sarcopenia is characterized by several muscle metabolic changes that occur within a lifetime, leading to the alteration of muscle strength, mass and quality [48]. The pathogenesis of sarcopenia is complex and involves inflammation [49], insulin resistance [50,51], ectopic lipid accumulation in skeletal muscle [52,53,54] and altered mitochondrial functions [55,56].

Consequently, as RA is associated with sarcopenia, it could be hypothesized that these abnormalities observed during aging and obesity also occur in RA patients and contribute to the consequences of RA on patient’s mobility and quality of life.

#### 3.2.1. Inflammation

RA is characterized by systemic and local inflammation in the synovial membrane of joints. This increased inflammation could be implicated in RA-related CV risk in RA patients. The paradox in the alteration in lipid profile was linked to inflammation. Indeed, Liao et al., showed that reduced hs-C-reactive protein (CRP) plasma level in RA patients was associated to an improvement of HDL cholesterol efflux capacity [57]. Thus, it can be postulated that the resolution of inflammation in RA could help to normalize HDL’s lipidome and, consequently, reduce CV disorders.

Elevated levels of CRP, and proinflammatory cytokines, such as IL-6 and TNF-α, may promote muscle loss [58] and were associated in older individuals with reduced muscle mass and strength [59]. It suggested that inflammation could also be involved in the progression of RA-related sarcopenia. Indeed, in a cohort of 457 RA patients, 45.1% presented myopenia and elevated CRP levels, suggesting that inflammation could induce sarcopenia [49]. About 20% to 30% of patients with RA have a decreased muscle mass [21,24,36]. The loss of muscle mass correlates with the activity and severity of inflammatory disease, and quality of life [28,29,30,36]. The role of inflammation in RA-related sarcopenia was further supported by the gain in lean mass following IL-6 inhibition with tocilizumab treatment [60]. Data on TNF-α antagonists are less consistent [60].

#### 3.2.2. Insulin Resistance

**Figure 2:**
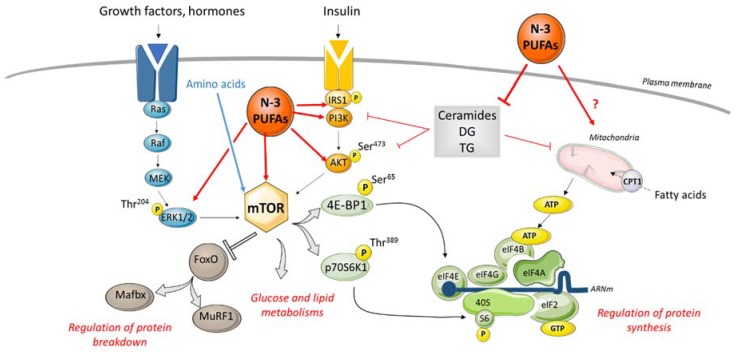
Modulation of muscle protein synthesis and degradation signaling by omega 3 fatty acids (FA). Several factors, such as insulin, amino acids, hormones, cytokines and growth factors can induce the activation by phosphorylation of PI3K/Akt and MAPK pathways. This leads to the activation of mTOR, which regulates glucose and lipid metabolism, activates protein synthesis and inhibits protein breakdown. Mitochondrial function is also a regulator of muscle protein synthesis by providing ATP. Muscle lipotoxicity is characterized by the accumulation of ceramides and diacylglycerols (DG) in muscles, inducing the dysregulation of protein synthesis through the inhibition of the PI3K/Akt pathway and mitochondria activity. If chronically induced, these events can lead to a decrease in skeletal muscle mass. Supplementation with omega 3 FA reduces lipotoxicity-induced muscle metabolism disorders. This effect is mediated by the increased mTOR activation by PI3K/Akt and MAPK pathways and the reduction of ceramides and DG content in muscle. Thus, supplementation with omega 3 FA could improve muscle protein turnover and counteract the loss of skeletal muscle related to lipotoxicity. No data are currently available about the effect of omega 3 FA on mitochondria metabolism during RA. CPT1: Carnitine-palmitoyl transferase 1; DG: Diacylglycerols; eIF: eukaryotic translation Initiation Factor; ERK1/2: Extracellular signal-Regulated Kinases 1/2; FoxO: Forkhead box O; IGFR: Insulin dependent Growth Factor-1 receptor; IR: Insulin receptor; IRS1: Insulin receptor substrate 1; Mafbx: Muscle atrophy F box protein; MurF1: Muscle RING Finger 1; n-3 PUFA: n-3 PolyUnsaturated Fatty Acids; PI3K: PhosphatidylInositol3-Kinase; p70S6K: p70 S6 kinase; Ser: serine residue; S6: S6 ribosomal protein; Thr: Threonine residue; 4E-BP1: 4E-Binding Protein 1.

The increased prevalence of insulin resistance [13] with RA is related to the action of both proinflammatory cytokines (IL-6, TNF-α) and glucocorticoids [17]. Insulin resistance is correlated to disease activity and the severity of the pathology [20] but not with all proinflammatory cytokines [61]. Moreover lower insulin-like growth factor-1 (IGF-1) levels were linked to reduced muscle cross-sectional area, reduced muscle density and a high severity of the RA [62]. In skeletal muscle, the stimulation of protein synthesis is strongly linked to the activation of mTOR protein and the initiation of protein translation (Figure 2) by insulin. Thus, alteration in insulin action leads to anabolic resistance, decreasing muscle protein synthesis and inducing muscle atrophy [63,64] Taken together, these data indicate that inflammation, in addition to other factors, could contribute to insulin resistance during RA and then in the alterations in muscle mass and function. Unfortunately, the mechanisms implicated remain unknown.

#### 3.2.3. Lipotoxicity

The accumulation of toxic lipid mediators is commonly defined by the term lipotoxicity and could be involved in the progression of sarcopenia. A lower rate of muscle protein synthesis was observed in obese rats after anabolic stimuli such as amino acids and insulin, defining the concept of anabolic resistance due to lipid overload [63]. In addition, Tardif et al., showed that muscle ectopic fat deposition could lead to anabolic resistance in obese sarcopenic old rats [64]. Thus, lipotoxicity could alter muscle metabolism but the mechanisms and cellular pathways are not completely understood. Lipotoxicity is particularly increased by SFA such as palmitate and mediated by the increased cellular levels of free-fatty acids (FFA), acyl CoA, triglycerides (TG), diacylglycerols (DG) and ceramides, contributing to the alteration of insulin sensitivity and protein synthesis, as observed in C2C12 cells [64,65,66]. Peterson et al., demonstrated that the treatment of C2C12 cells with palmitate also induced apoptosis [67]. Effects of SFA on muscle development were also described. The treatment of mouse muscle cells with palmitate increased cell death by inducing caspases and decreased differentiation by reducing myogenin expression, leading to a reduction of the regenerative capacity of muscle cells [68]. No link has been demonstrated between lipotoxicity in skeletal muscle and the loss of muscle function during RA. Our group recently showed that muscle atrophy in rats with collagen-induced arthritis was associated with ectopic lipid accumulation and reduced mitochondrial DNA copy number in skeletal muscle, decreased PPARγ mRNA expression in the perigonadal adipose tissue suggesting a reduced adipogenic capacity [69]. Increased expression of muscle atrophy F-box protein (MAFbx) mRNA, an enzyme implicated in protein degradation, and a decreased expression of myocyte differentiation protein (MyoD) mRNA, implicated in protein synthesis and muscle regeneration were also noted. This work confirmed previous results which highlighted a decrease of muscle regeneration in arthritic rats which was partly related to a decrease of MyoD and myogenin mRNA levels in skeletal muscle [70,71]. Thus, the reduced adipogenic capacity of adipose cells and skeletal muscle mitochondrial capacity are probably involved in the intramuscular lipid accumulation, activation of protein catabolism, and inhibition of myogenesis, which ultimately lead to skeletal muscle fiber atrophy during RA.

#### 3.2.4. Mitochondrial Dysfunction

The maintenance of muscle mass and function is closely related to the quality of mitochondria activity and the production of energy in the form of ATP. Age-related sarcopenia is associated with mitochondrial dysfunctions related to a decrease in organelle number, enzymatic activities (citrate synthase, cytochrome c oxidase), FA β-oxidation and mitochondrial DNA copy number. Furthermore, the increase in oxidative damages in skeletal muscle with aging could be linked to an excessive production of ROS by the mitochondria. Oxidative damages may aggravate mitochondrial dysfunctions and further accelerate the progression of sarcopenia. The hypothesis that mitochondrial dysfunctions could be involved in sarcopenia in RA patients could be drawn from the observation that oxidative stress was linked to chronic inflammation and RA [72]. The correlation between ROS production and the loss of handgrip strength in older women [73], the contribution of oxidative stress to skeletal muscle dysfunction in an animal models of RA [74] further corroborated this hypothesis. Mitochondrial dysfunctions during RA were proposed to be mediated by IL-17. Hence, inflammation could impair mitochondrial functions through the modulation of IL-17 production by Th17 lymphocytes. It is known that IL-17 plays a critical role in the development and the progression of RA. It was also demonstrated that the production of IL-17 in synovial fibroblasts induced morphological changes of the organelle, respiratory dysfunctions and activated mitophagy [75]. Recently, our group showed that RA was associated to a decrease of mtDNA content and cytochrome c oxidase activity in skeletal muscle [76]. The decline in mitochondrial FA β-oxidation could also contribute to ectopic lipid accumulation and the RA-related increase in adiposity and metabolic disorders. These events will contribute to further aggravate all the mentioned alterations, amplifying the accumulation of ectopic fat and the progression of anabolic resistance [77,78]. However, the links between mitochondrial dysfunctions in skeletal muscle, lipitoxicity, IL-17 and RA remain unexplored.

Alterations in inflammatory status, insulin resistance, lipid homeostasis and mitochondrial activity appear as a key factors that could be involved in sarcopenia during RA. Considering the accumulation of experimental evidence showing the health effects of omega 3 FA, this raises the question of the role of these essential nutrients to limit comorbidities induced by RA and the consequences on skeletal muscle.

## 4. Consuming Omega 3 to Improve Muscle Health in RA?

Reports of dietary intake in Western countries clearly pointed out an excessive consumption of SFA and a deficit in the intake of polyunsaturated FA (PUFA), notably of the omega 3 family [79,80]. SFA are pro-inflammatory and increase CVD risk and mortality, whereas other FA, such as omega 3 FA, are anti-inflammatory [5,81]. Dietary guidelines from the World Health Organization and in North America claim that daily intake of lipids should cover 20–35% of daily energy intake (DEI) [82,83]. The value has been increased to 35–40% in France [80]. These guidelines also propose recommendations for the intake of omega 3 FA. The intake of α-linolenic acid (ALA), the precursor for the synthesis of EPA and DHA should cover 0.5–1% of DEI (that corresponds to approximately 0.8–1 g/day). However, because of the poor bioconversion of ALA to EPA and DHA, intake recommendations for EPA and DHA were also claimed, corresponding to at least 0.25 g/day [80,82,83]. In clinical trials targeting CVD or RA, supplementations generally provided more than 1 g/day and frequently reached 2–4 g/day [8,84]. The major nutritional sources of EPA and DHA are fish oil and to a lesser extent, seal oils, krill and algaes. Additionally, many dairy products were developed to be enriched with omega 3 FA and constitute then a promising source [85]. If the recommended intake of omega 3 FA for the general population could be easily achieved by fish consumption, the prescription of more than 1 g/day to patients, for example, with coronary heart disease, requires enriched supplements. These supplements may include natural triglycerides (from fish oil), re-esterified triglycerides, phospholipids, purified EPA or DHA ethyl esters. Caution should be taken when choosing the supplement. It has been described that concomitantly to a decrease in plasma triglycerides, both HDL-c and LDL-c levels were increased after omega 3 supplementation even if this effect seemed more related to DHA than EPA intake [86]. The use of larger doses of natural oils can also result in an elevated intake of vitamins, SFA and cholesterol, depending on the origin of the oil. The form of the supplementation or prescription should be discussed with the pharmacist, the nutritionist or the physician to check the content of the products and the best dosage.

### 4.1. Omega 3 and Inflammation in RA

EPA and DHA are the most bioactive FA of the omega 3 family and could be metabolized in anti-inflammatory metabolites called resolvins [8,87]. Arachidonic acid (AA) an omega 6 FA is a precursor of the major part of inflammatory mediators called eicosanoids, including prostaglandins, leukotrienes and thromboxanes. The eicosanoids from AA have a high biological activity and the increase in their production contributes to the risk of thrombus, atheroma, allergy, and inflammatory disorders. On the contrary, omega 3-derived metabolites decrease the pathogenesis of cardiovascular and inflammatory disorders [88]. The biosynthesis of omega 3 and omega 6-derived molecules is possible through enzymatic reactions that are commons. Thus, EPA and DHA are in competition with AA for the biosynthesis of their respective bioactive derivatives [6]. The PUFA composition of cell membranes is strongly dependent on dietary intakes. The EPA and DHA that are eaten replace the AA in membrane phospholipids. Considering, the high omega 6 to omega 3 ratio in a typical Western diet, any increase of the amount of omega 3 is supposed to reduce the synthesis of pro-inflammatory eicosanoids from AA and thus, to negatively regulate inflammation [6].

A study performed in Fat-1 mice, which exhibit a spontaneous conversion of omega 6 FA to omega 3 FA, has identified a promising effect of omega 3 in RA. Indeed, arthritis was attenuated in Fat-1 mice compared to controls, especially by reducing ankle joint inflammation and bone damages and a reduced production of IL-17, IL-6 and IL-23 [89]. A dietary supplementation with omega 3 using fish oil in a mouse model of RA decreased the plasma levels of IL-6, IL-10, IL-12, TNF-α, prostaglandin PGE_2_ and thromboxane TXB_2_ [90]. Other results suggested that these effects could be mediated by the production of resolvins. Indeed, in RA mice fed with fish oil, the levels of pro-inflammatory derivatives were reduced in favor of the production of resolvins [87]. The intake of 2.4 g/day of EPA and DHA in patients with inflammatory arthritis increased the plasmatic concentrations of resolvins, the amount of resolvin E2 in synovial fluid and reduced pain and inflammation [91].

In humans, these results were confirmed in a cohort of 3042 healthy subjects. Inflammatory markers, such as CRP, IL-6 and TNF-α levels, were decreased by the consumption of fish oil [92].

Some reports showed that omega 3 could have a beneficial effect on RA severity and its related comorbidities. The consumption of fish oil during 3 years in early RA patients reduced the concentration of the omega 6 derived inflammatory mediators, such as thromboxane TXB_2_ and PGE_2,_ in serum [93]. Omega 3 FA supplementation in RA patients reduced IL-1β plasmatic levels [94,95]. Some studies have also shown that a diet enriched in omega 3 FA can prevent the apparition of RA or its evolution. Fish consumption was associated to a decreased risk of RA [96]. Gan et al., shown that supplementation of RA patients with omega 3 FA reduced circulating autoantibodies, which are closely related to the progression of the disorder [97]. Moreover, high doses of fish oil (54 mg/kg EPA and 36 mg/kg DHA per day) reduced pain, morning stiffness and improved tender joint count and swelling joint count [95]. It improved also the remission when associated to conventional disease-modifying DMARDs [98]. In addition, the concentration of EPA in serum was associated with a better response to anti-TNF therapy [99]. Taken together, these results suggested that omega 3 FA have a favorable effect on inflammation and pain in RA, that can be, at least partially, explained by the generation of omega 3 derived lipid mediators [100,101]. Yet, counteract inflammation and pain by omega 3 supplementation or intake could improve mobility, and thus muscle health of RA patients.

### 4.2. Omega 3 and CV Risk

#### 4.2.1. Effects on Lipid Profile

In vivo studies have shown that EPA and DHA improved plasma lipid profile in patients with a metabolic syndrome. Indeed, in dyslipidaemic, obese and insulin resistant subjects, omega 3 FA had limited effects on total cholesterol and LDL-c but increase HDL-c level [102]. DHA and EPA increased LDL particle size in hypertensive type 2 diabetes patients and with hypertriglyceridemia respectively, contributing to the protection against CVD [103,104]. Moreover, it was showed that omega 3 FA reduced circulating oxidized LDL and improved endothelial function in patients presenting a high risk for CVD [105,106,107]. Omega 3 FA directly replace proinflammatory omega 6 FA in biological membranes. Thus, the improvement of the phospholipid composition of HDL particle could increase their atheroprotective activity and thus affect the progression of CVD in RA patients [108].

#### 4.2.2. Effects on CVD

Even if it is commonly accepted that omega 3 FA are protective against CV risk, the impact of their intake or supplementation on CVD remains controversial. Indeed, several studies raised a doubt about the beneficial effect of omega 3 FA supplementation on mortality and cardiovascular-related outcomes [84,109]. However, a systematic literature search showed that the consumption of EPA and DHA could be associated with a reduction of coronary heart diseases [110]. A meta-analysis of 2890 articles, including 16 trials with 861 participants, showed that a daily supplement of omega 3 FA significantly improved endothelial function by increasing Flow Mediated Dilatation (FMD). This effect was more evident in participants with a poor health status [111]. In addition, in hypertensive patients with hypertriglyceridemia and high cardiovascular risk, an interventional study showed that supplementation with omega 3 FA improved arterial stiffness and endothelial function [105]. Tousoulis et al., demonstrated that a daily administration of omega 3 FA (2 g/dose, 46% EPA-38% DHA) induced an improvement in endothelial function, arterial stiffness and inflammatory status in 29 patients with a metabolic syndrome without CVD nor inflammatory disease [112]. Finally, increased intakes of omega 3 FA in RA patients were associated with an improvement in arterial stiffness [103]. Taken together, these data suggest that omega 3 FA could have an effect on CV functions and thus on CVD outcomes in a population with a high CV risk, but not in primary prevention, as shown in a randomized trial by Manson et al. [109]. Yet, CVD constitute a factor of disability and loss of mobility, which affect muscle health. The prevention of CVD with omega 3 FA supplementation could thus have promising effect on muscle health in RA patients.

### 4.3. Omega 3 and Muscle Metabolism

In parallel to their effect on lipid profile, it has also been shown that omega 3 FA could prevent the development of lipotoxicity in skeletal muscle (Table 1). Several in vitro studies have investigated the effect of omega 3 FA on lipotoxicity and its consequences on muscle metabolism. Omega 3 FA could decrease palmitate-induced lipotoxicity in C_2_C_12_ muscle cells. Hence, treatment of these cells with EPA or DHA reduced the accumulation of toxic lipid mediators such as DG and ceramides and preserved the activation of insulin signaling [9,65].

Other studies have shown that supplementation with omega 3 FA can be protective for the preservation of insulin response in skeletal muscle. Observational studies in adults have showed that circulating EPA levels were inversely correlated to insulin resistance [113,114]. Nigam et al., demonstrated in 353 subjects with metabolic syndrome, that high plasma levels of EPA and DHA reduced metabolic syndrome and insulin resistance [113]. This effect was even highlighted in the Inuit population which has a high level of fish consumption [114]. An interventional study conducted in healthy adults treated with dexamethasone to induce insulin resistance, showed that the intake of fish oil (1.1 g EPA and 0.7 g DHA per day) decreased insulin plasma levels [115]. The improvement in insulin sensitivity and the inhibition of the accumulation of toxic lipids may depend on modifications at the level of muscle lipid homeostasis induced by omega 3 FA (Table 1 and Figure 2) [116,117,118].

An impaired mitochondrial function led to an altered β-oxidation rate of FA, resulting in the accumulation of ectopic fat in peripheral tissues such as skeletal muscle [116]. Treatment of human skeletal muscle cells with EPA reduced lipid accumulation, increased lipolysis and oxidation of FA [117]. In rats fed with a high-fat diet rich in fish oil, an enhancement in mitochondrial respiratory uncoupling was observed in hind leg muscle compared to rats fed with a standard high-fat diet [118]. This effect was probably related to an increased expression of the mitochondrial uncoupling protein 3 (UCP3) [119]. Moreover, the dietary supplementation with omega 3 FA increased CPT-1 expression and activity in rat skeletal muscle, indicating an increase in FA β-oxidation (Figure 2) [77,120]. Thus, omega 3 FA could increase lipid oxidation to limit or prevent the production of lipotoxic mediators implicated in the development of CVD and sarcopenia.

Taken together, these results suggest that supplementation with omega 3 FA could reduce lipotoxicity and thus protect from insulin and anabolic resistance (Figure 2).

Some evidences also proposed that omega 3 FA could improve muscle protein metabolism in stress conditions (Table 1). Feeding C57BL/6 mice during 8 weeks with a DHA-enriched diet led to the preservation of tibialis anterior muscle mass after 48 h fasting [127]. Moreover, Smith et al., showed that omega 3 FA supplementation in healthy older adults with anabolic resistance improved muscle protein anabolic response and muscle protein rate through an increase in the phosphorylation of mTOR^Ser2448^ and p70S6K^Thr389,^ signaling (Figure 2) [128,129]. Beneficial effects of omega 3 FA on muscle metabolism were also described in a situation combining inflammation and lipotoxicity. EPA improved the regenerative capacity of skeletal muscle cells exposed to SFA and inflammation [58]. Yet, as mentioned previously, EPA reduced lipotoxicity, suggesting that its favorable effect on muscle metabolism could be due to a protective effect against abnormalities in lipid homeostasis [68,117]. The degradation of muscle protein is increased by inflammation and could be regulated by treatment with omega 3 FA (Table 1). In myotubes, EPA inhibited the effect of TNF-α on muscle proteolysis [124]. DHA was also found to inhibit muscle protein degradation in C_2_C_12_ [125]_._ These results were confirmed by Shin et al., who showed that DHA reduced myotube degradation in an in vitro model of muscle atrophy [126].

In RA, only one study showed that EPA administration could prevent the increase of TNF-α and atrogin-1 induced by the arthritis in muscle [71]. These results remain to be confirmed.

Thus, it can be postulated that supplementation with omega 3 FA during RA could reverse muscle lipotoxicity and reduce anabolic resistance. These effects would be crucial factors leading to a decrease in the development of comorbidities and mortality (Table 1 and Table 2).

## 5. Conclusions

In conclusion, RA is associated with an increased morbi-mortality, mainly due to comorbidities and cardio-metabolic disorders, such as sarcopenic obesity and CVD (Figure 1). Alterations in lipid homeostasis and in the quality of dietary lipids are potential causative elements of these abnormalities which share common physiopathological mechanisms.

Thereby, a nutritional approach including supplementation with omega 3 FA should be proposed to alleviate lipotoxicity in the context of aging, obesity and chronic inflammatory diseases such as RA. The preservation of skeletal muscle health will therefore contribute to prevent the occurrence of CVD and metabolic disorders. This hypothesis remains to be demonstrated.

However, the protective effects of omega 3 FA on the incidence of CVD are still debated [84,143]. Several studies showing a favorable effect were conducted with high doses of omega 3 FA. Such doses could be difficult to reproduce in daily practice. The effects and interactions of high doses of omega 3 FA are unclear and still need to be evaluated [144].

## 6. Perspectives: Multimodal Approaches

Further work should be performed to explore the relevance of multimodal strategies, including the consideration of other nutrients such as protein but also physical activity and/or specific medications (Table 2). Physical activity is known to improve TG and HDL-c serum levels [132], and helps to preserve lean body mass, muscle anabolic response and muscle function in RA patients [136,137,138]. On the other hand, an optimized protein intake seemed to have beneficial effects on muscle protein metabolism, muscle mass and quality. Biologic therapies targeting proinflammatory cytokines decrease inflammation and thus, improve cardiovascular risk, metabolic disorders and body composition. Indeed, in RA patients, treatment with TNF-α inhibitors was associated with a decrease in the incidence of CVD [145], an increase in HDL-c levels and an improvement of the quality of phospholipids-HDL. The inhibition of IL-6 was shown to restore skeletal muscle mass with a favorable cardio metabolic profile in RA patients, without any change in fat mass [60]. All of these data clearly suggest that the protective effect of omega 3 FA in inflammation and CVD can be synergistic with physical activity and biologic therapies to increase muscle health, particularly in RA (Table 2). This hypothesis still needs observational and interventional studies. Finally, because cardiometabolic comorbidities could share common pathophysiological pathways in the context of rheumatic diseases, it appears conceivable that the nutritional and interventional strategies proposed to RA patients could also be tested in patients with OA and Psa.

## Figures and Tables

**Table 1:** Omega 3 and muscle metabolism.

Model	Intervention	Results	References
**Lipid Metabolism**			
**In Vitro**			
C2C12 muscle cells	50 µM EPA or DHA-16 h	Reduction of TG, DG and ceramide content	Pinel et al., 2016 [9]
**In Vivo**			
Rats fed with a HFD (*n* = 8)	Fish oil supplementation for 6 weeks	Increase of mitochondrial respiratory uncoupling in hind leg muscle	Cavaliere et al., 2016 [118]
Wistar rats with a HFD (*n* = 6)	Fish oil supplementation for 10 weeks	Increase of CPT1 expression and activity	Power et al., 1997 [120]
**Carbohydrate metabolism**			
**In Vitro**			
C2C12 muscle cells	500 µM palmitate + 30 µM DHA-16 h	Restoration of insulin response altered by palmitate-treatment	Capel et al., 2015 [65]
C2C12 muscle cells	50 µM EPA treatment-180 min	Increase of 2-DOG uptake	Figueras et al., 2011 [121]
**In Vivo**			
Rat with spontaneous type 2 diabetes (*n* = 10)	EPA 0.5 g/kg for 28 days	Increase of GLUT4 mRNA in skeletal muscle	Figueras et al., 2011 [121]
Male ob/ob mice (*n* = 16)	6% of lipid content was provided by omega 3 for 5 weeks	Increase of GLUT4 mRNA and phosphorylation of IRS-1 and Akt in skeletal muscle	González-Périz et al., 2009 [122]
Human skeletal muscle cells (vastus lateralis)	0.6 mM EPA retreatment-24 h	Increase of glucose transport in response to 100 nM insulin-15 min	Aas et al., 2006 [123]
**Protein metabolism**			
**In Vitro**			
C2C12 muscle cells	75 mM palmitate + 50 µM EPA pretreatment-1 h	Increase of muscle regeneration capacities	Saini et al., 2017 [68]
C2C12 myotubes	50 µM EPA treatment-24 h	Decrease of 3H-Phe muscle release induced by TNF	Mirza et al., 2016 [124]
C2C12 muscle cells	300–600 µM DHA and EPA-24 h	Inhibition of muscle protein degradation	Wang et al., 2013 [125]
C2C12 muscle cells overexpressing aggregation-tau protein	DHA 100 µM-4 h	Reduction of myotube degradation by inhibiting S26 proteasome activity	Shin et al., 2017 [126]
**In Vivo**			
C57BL/6 mice (*n* = 20)	8 weeks DHA enriched-diet	Tibialis anterior preserved after a 48 h-fasting	Deval et al., 2016 [127]
Wistar collagen-induced arthritis rats (*n* = 18)	12 days EPA oral administration	Prevention of TNF-α and atrogin-1 increase induced by arthritisAttenuation of the gastrocnemius atrophy and of the increase of MuRF1 induced by RA	Castillero et al., 2009 [71]

Omega 3 can modulate muscle lipid, carbohydrate and protein metabolisms. Indeed, several studies showed that omega 3 FA could improve muscle lipotoxicity by increasing mitochondrial activity. This could induce an improvement of muscle insulin sensitivity as insulin response and glucose uptake. Thus, in a situation of lipotoxicity, muscle protein metabolism could be protected by omega 3, as proteolysis was decreased and muscle mass was preserved. Currently, no data are available about the effect of the supplementation with omega 3 FA on lipotoxicity in RA.

**Table 2:** Protective effect of nutrition, therapy and physical activity on lipid metabolism disorders and CV diseases.

	Dietary Lipids	Physical Activity	Therapy
Lipid profile	Omega 3 fatty ↗ HDL-c levels [102]	More exercise is associated with smaller HDL-P, fewer large HDL-P and reduced mean HDL-size [130]	TNF-α inhibitors ↗ TC and HDL-c levels [131]
	Omega 3 fatty ↘ circulating oxidized LDL-c [105,106,107]	Correlation between the intensity of physical activity and HDL levels [132]	3 months anti-TNF-α treatment ↘ TC levels [133]
	DHA and EPA ↗ LDL particle size in hypertensive type 2 diabetes patients and with a hypertriglyceridemia [103,104]	Inverse correlation between the intensity of physical activity and TG and LDL levels [132]	TNF-α inhibition ↗ anti-inflammatory properties of HDL-c [134]
Muscle lipotoxicity	50µM EPA or DHA ↘ TG, DG and ceramides content [9]	Endurance training ↗ lipid turnover and improve lipid droplets quality [135]	
	EPA ↗ muscle regeneration capacity of C_2_C_12_ muscle cells exposed to palmitate [68]		
Muscle mass and function	Omega 3 during 8 weeks ↗ protein anabolic response in healthy adults [129]	Acute resistance exercise preserved lean body mass, muscle anabolic response and muscle function [136,137]	
	Omega 3 supplementation ↗ muscle protein rate and phosphorylation of mTOR^Ser2448^ and p70S6K^Thr389^ [128]	Long term training program combining strength and endurance ↗ muscle functions [138]	
	DHA ↘ muscle protein degradation in C_2_C_12_ [125,126]		
CV diseases	2g omega 3 (46% EPA-38% DHA) ↗ endothelial function and ↘ arterial stiffness [112]	Exercise is associated with a reduced vascular stiffness in RA [139]	3 months anti-TNF-α treatment improved blood pressure in RA patients [133]
	Omega 3 ↗ endothelial function in 16 patients with hypertriglyceridemia [105]	Resistance exercise improves endothelial function in type 2 diabetes subjects [140]	3 months anti-TNF-α treatment improved endothelial function in RA patients [141]
	Omega 3 fatty acids ↗ FMD [111]	Moderated-vigourous physical activity ↘ FMD and blood pressure and not affected vascular function [142]	TNF-α inhibitors ↘ the incidence of CV diseases [86]

Nutrition could have protective effect against perturbations in lipid profile, muscle lipotoxicity, sarcopenia and CV diseases that occur in RA patients. Indeed, an omega 3 supplementation improve lipid profile by increasing HDL-c plasma levels and decreasing TC and LDL-c levels. Moreover, in vitro studies showed that EPA and DHA decreased muscle lipotoxicity and improved muscle protein metabolism, leading to a decrease of CV dysfunctions. This nutritional approach could have a synergic effect with therapy, since TNF-α inhibitors improved lipid profile and decreased CV risk, or with physical activity, which improved lipid profile, muscle metabolism and CV functions. However, no effects of therapy on muscle metabolism has yet been investigated. DG: Diglycerides; DHA: Docosahexaenoic acid; EPA: Eicosapentaenoic acid; HDL: High-density lipoprotein-cholesterol; LDL-c: Low-density lipoprotein-cholesterol; ox-LDL-c: oxidized low-density lipoprotein-cholesterol; RA: Rheumatoid Arthritis; TC: Total cholesterol; TG: Triglycerides; TNF-α: Tumorous Nuclear Factor α.
